# Polymyalgia rheumatica: diagnosis, prescribing, and monitoring in general practice

**DOI:** 10.3399/bjgp13X667231

**Published:** 2013-04-29

**Authors:** Toby Helliwell, Samantha Lara Hider, Christian David Mallen

**Affiliations:** Arthritis Research UK Primary Care Centre, Primary Care Sciences, Keele University, Keele.; Arthritis Research UK Primary Care Centre, Primary Care Sciences, Keele University, Keele.; Arthritis Research UK Primary Care Centre, Primary Care Sciences, Keele University, Keele.

**Keywords:** disease management, general practice, polymyalgia rheumatica, prednisolone

## Abstract

**Background:**

Polymyalgia rheumatica (PMR) is a common rheumatological disorder of older patients. The majority of UK patients are diagnosed and managed exclusively in general practice. In primary care, it has been shown that there is wide variation in practice, and established diagnostic criteria are infrequently used.

**Aim:**

This study aims to investigate the diagnostic processes, management, and monitoring of patients with PMR in UK primary care.

**Design and setting:**

This is a retrospective cohort study set in primary care.

**Method:**

Data were extracted from two interlinked primary care databases from north Staffordshire. Patients with PMR were identified using Read Codes and the relevant investigation, prescription, and consultation data were extracted and reviewed.

**Results:**

Three hundred and four patients’ records were analysed. Documentation of symptoms leading to a diagnosis of PMR was found in 248 records (81.6%). A documented process of exclusion of relevant differential diagnoses was demonstrated in 68 (22.4%) patients. The mean initial dose of prednisolone was 21.5 mg. Referral to specialist care was made for 135 (44.4%) patients. Gastric prophylaxis was prescribed in 85 (28.0%) cases. Osteoporosis prophylaxis was prescribed to 183 patients (60.2%); 12 patients (3.9%) developed osteoporosis and 56 (18.4%) developed gastric symptoms that led to GP consultation.

**Conclusion:**

The management of PMR in general practice could be optimised. Identified areas for improvement include clear documentation of a process of exclusion of other diagnoses, and prophylaxis for potential treatment complications, including osteoporosis and gastric symptoms.

## INTRODUCTION

Polymyalgia rheumatica (PMR) was first described by Bruce in 1888,^1^ and is a common inflammatory rheumatological disorder affecting those aged >50 years,^2^ with a peak incidence in the UK of 22.9 per 10 000 patient-years in the age range of 70–79 years.^3^ The reported incidence varies, from a low of 12.7/100 000 in northern Spain,^4^ to a high of 112/100 000 in Norway.^5^ It carries a lifetime risk of 2.4% in females and 1.7% in males.^6^ In the UK, a full-time GP working in a practice with a list size of 10 000 patients will see, on average, five cases of PMR per year,^7^ and it has been shown that over 80% of patients are exclusively managed in primary care.^8^ Studies have suggested that the primary care management of PMR varies widely,^9^ and that available classification criteria are rarely used.^8^ Some studies have also inferred poor diagnostic accuracy of PMR in general practice.^10^ As previously described, the majority of patients are managed exclusively in general practice,^8^ yet the majority of research on PMR is based on patients recruited from secondary care settings, despite the call, in the past, for more primary-care-based research.^11^ Patients referred for specialist review are likely to have more comorbidities, be younger, and have a normal or near-normal erythrocyte sedimentation rate (ESR),^12^ and so are less likely to represent the typical patient with PMR seen by GPs.

No diagnostic gold standard test exists for PMR, and so clinicians have to rely on existing classification criteria, laboratory findings, and response to treatment, to make a diagnosis, although controversy still exists as to the defining characteristics of the illness.^13^

Low-dose corticosteroids are an effective treatment for many patients, often resulting in a striking improvement of symptoms.^14^ This response to corticosteroids is considered an important diagnostic feature of PMR.^15^,^16^ Many conditions, including malignancy, can mimic some of the symptoms, signs, and laboratory findings of PMR, and an attempt should be made to exclude these conditions before a diagnosis of PMR is formally made.^15^,^16^ Additionally, diagnosis can be made more difficult, as some of the symptoms of these illnesses may also improve initially when treated with low-dose corticosteroids. Corticosteroids are often well tolerated; however, long-term corticosteroid treatment is associated with well-recognised side effects and complications, such as osteoporosis.^17^ PMR is one of the most common rheumatological indications for long-term corticosteroids in older females,^18^ and so for patients with PMR, these potential complications and side effects represent a significant risk. Therefore, regular assessment, prevention, and treatment of these complications should form part of the standard management of this common rheumatological disease.

PMR remains an area that is under-researched, specifically within primary care where the majority of patients are exclusively managed. The aim of this study was to further investigate how GPs diagnose and manage PMR. Additional specific objectives included a comparison of practice with current national guidelines,^15^ and an assessment of the occurrence and management of common and potentially preventable corticosteroid complications such as osteoporosis, peptic ulceration, dyspepsia, and reflux disease. Current guidance suggests that calcium and vitamin D should be offered to all patients on long-term corticosteroids, with the addition of a bisphosphonate in high-risk groups (T score on DEXA [dual-energy X-ray absorptiometry] scan below −1.5, previous fragility fracture, and age >65 years);^15^,^19^ however, to date, there have been no specific guidelines regarding gastric prophylaxis for patients on long-term corticosteroids.

**How this fits in**

Polymyalgia rheumatica (PMR) is the most common inflammatory rheumatological disorder seen in older patients, the majority of whom are managed exclusively by GPs. Little research has been undertaken in a primary care setting. This study outlines the current UK diagnostic and management practice in a large primary care-based cohort of patients with PMR, and highlights where clinical practice could be improved and further research is needed.

## METHOD

This study was undertaken using two interlinked primary care databases at the Arthritis Research UK Primary Care Centre at Keele University. The Consultations in Primary Care Archive (CiPCA) is a database that contains patient consultation data, which includes the practice code, date of consultation, diagnostic Read Code, type of encounter (new or follow-up), location (home visit, surgery, telephone), and free text, which is limited to 255 characters. The Prescribing in Primary Care Archive (PiPCA) database consists of prescribing data (type of medication, date of issue, prescription instructions, quantity issued) and investigation data (test ordered, date of request, results of investigation). This information was retrieved from 14 general practices in north Staffordshire that contribute data to the databases. Each patient is assigned a unique identifier, which allows the relevant prescribing and investigation data to be linked between the two databases. These databases were chosen, as they represent local practices to Staffordshire and participating practices undergo regular training in coding and data entry, quality assessment, and audit, with regular feedback to ensure the data remains of a high quality.^20^ Practices contributing to this dataset are broadly representative both of local and national (for example, GPRD [General Practice Research Database] databases.^21^

Read Codes are a hierarchical clinical classification coding system used in UK general practice. A search by Read Code through the databases for patients diagnosed with polymyalgia rheumatica (Read Code N20) or polymyalgia (Read Code N20-1) from the beginning of 1999 to the end of 2006 was undertaken. All cases identified were included in the initial data review.

Using recent British Society of Rheumatology and British Health Professionals in Rheumatology guidelines for PMR,^15^ areas for further review relevant to primary care were identified. These areas included suggested diagnostic work-up, management, and follow-up subsequent to diagnosis. Relevant diagnostic and management data were extracted by hand and rechecked by a single researcher, then and entered onto a data-extraction document developed specifically for this study. This was coded and the data entered onto a separate database. This included initial and ongoing corticosteroid treatment, prescription of osteoporosis and gastric prophylaxis, initial ESR, and evidence of other laboratory investigations that might be used to exclude other causes of symptoms. The dates of prescriptions and investigations could be linked to specific consultations between the two databases, using the unique patient identifier.

Formal referral data are not stored on the CiPCA database and therefore it was necessary to rely upon separate coding of the referral or documentation in free text. Potential associations between specialist referral and age, sex, and ESR were investigated. Using the prescribing data on the PiPCA database, free text, or morbidity codes, an assessment of the presence or development of corticosteroid complications could be made. For osteoporosis, the diagnostic Read Code, DEXA scan result, or documentation in free text was used.

Patients were classified as developing gastric symptoms if a new diagnostic Read Code was added, a first prescription of proton pump inhibitor (PPI) or H2 antagonist was found, or new symptoms (for example, gastritis, dyspepsia, or gastro-oesophageal reflux) were documented in free text. The presence of any of these prior to first PMR diagnosis classified patients as having a pre-existing gastric comorbidity. These specific complications of treatment were chosen as they have a clear potential for prevention. Any evidence suggesting that an attempt had been made to exclude other causes for the presenting symptoms was considered. This included any relevant imaging or blood work-up that might suggest that a process of exclusion had occurred.

Current guidance suggests early referral for specialist review for patients who are aged <60 years,^15^ and so this age limit was used in assessing associations between age and referral. To assess which patients achieved the latest diagnostic guidelines, only complete records that had both a documented ESR and a clear and complete prescription of corticosteroids at diagnosis were used. Each complete record was reviewed by hand and analysed and compared to current guidance on documentation of relevant symptoms, appropriate diagnostic tests, correct initiating dose of corticosteroid, and response to initial treatment.

An expected response to corticosteroid treatment in PMR is a global improvement of ≥70% within a week of commencement.^15^ However, it is not usual in general practice to make formal assessments of patient-reported global health, and so a significant response was considered to be one that justified the diagnosis, resulting in continued treatment.

### Statistical analysis

Statistical analysis was performed using SPSS for Windows 15.0. Simple descriptive statistics (mean, median, standard deviation [SD]) were used to describe the data. Associations were investigated using χ^2^ tests, with *P*-values reported.

## RESULTS

**Figure 1:**
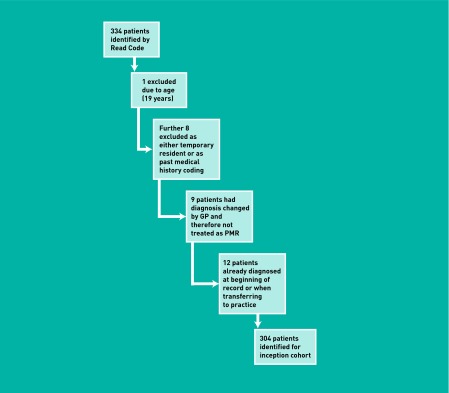
***Creation of the inception cohort.***

A total of 334 patients were identified from the CiPCA database with a Read Code for polymyalgia rheumatica (N20) or polymyalgia (N20-1) between January 1999 and December 2006. Thirty patients were excluded, leaving a final cohort of 304 for data analysis (Figure 1). Table 1 shows the baseline demographics of the study population.

**Table 1:** Baseline demographics of primary care PMR cohort

**Demographic**	**Median (IQR)**	***n* (%)**
Age at onset of symptoms, years	73 (66–80)	
<50		7 (2.3)
50–59		18 (5.9)
60–69		86 (28.3)
70–79		117 (38.5)
≥80		76 (25.0)
Total		304
≥65		252 (82.2)

**Sex**		
Female		229 (75.3)

Number of consultations, first PMR entry to last	9 (4 to 17)	–

Duration of treatment with corticosteroids, months^a^	14 (5 to 29)	–

IQR = interquartile range. SD = standard deviation.

a All patients were treated with corticosteroids, exclusively with prednisolone except in one case where the patient was treated with intramuscular methylprednisolone. However, the response to treatment was poor and so the patient was changed to oral prednisolone, resulting in a good response to treatment.

Documentation of the symptoms used to make a diagnosis was demonstrated in 248 (81.6%) cases, with the most common primary symptom being bilateral shoulder pain (45.7%) and myalgia being the second most common symptom (Table 2). A good response to initial steroids was seen in 221 patients (72.7%), and evidence of a process of diagnostic exclusion was seen in 68 cases (22.4%). The mean initial dose of prednisolone was 21.5 mg (SD = 8.336 mg), with the most frequent starting dose being 15 mg (found in 32.6% of patients). The mean ESR at diagnosis was 51 mm/hour (SD = 27.459 mm/hour), with 11.8% having a normal ESR (<20 mm/hour). Table 2 summarises these results.

**Table 2:** Diagnosis and initial management of PMR patients in primary care

**Diagnostic parameter**	

Symptom documentation, *n* (%)	248 (81.6)

**Specific PMR symptoms:^a^ primary symptom documented, *n* (%)**	
Bilateral shoulder pain	139 (45.7)
Hip girdle	9 (3.0)
Myalgias	32 (10.5)
Morning stiffness	8 (2.6)
Other	60 (19.7)
None documented	56 (18.4)
Total	304
Exclusion of other causes: yes, *n* (%)	68 (22.4)
Initial steroid dose, mean (SD), mg	21.5 (8.336)

**Response to corticosteroids, *n* (%)**	
Good response	221 (72.7)
Poor/no response	12 (3.9)
Unclear from records	71 (23.4)
Total	304
Time to initial follow-up, mean (mode), weeks	1.88 (1.0)

**ESR, mm/hour**	
Mean (SD)	51 (27.459)
Range	4 to 12]
Normal (<20 mm/hour), *n* (%)	36 (11.8)

ESR = erythrocyte sedimentation rate. SD = standard deviation.

a Specific PMR symptoms as defined by the BSR/BHPR guidelines.^15^

One hundred and thirty-five patients (44.4%) with PMR were referred for specialist review. During the course of treatment, 4% developed osteoporosis (identified by free text, DEXA scan result, or formal diagnostic entry) and 18.4% developed dyspeptic symptoms requiring GP consultation. Gastric prophylaxis was prescribed to 28.0% of the cohort, and this was significantly associated with a history of, or previous prescription of medication for treatment of, dyspepsia (χ^2^ = 100.797; *P*<0.001).

One hundred and eighty-three patients (60.2%) of the cohort received a prescription for osteoporosis prophylaxis (calcium + vitamin D supplementation, with or without bisphosphonate). This was found to be significantly associated with referral to secondary care (χ^2^ = 21.656; *P*<0.001). No associations were found between age or inflammatory burden (as measured by raised ESR) and secondary care referral.

To compare with current guidance,^12^ a complete record was required. Two hundred and thirty-three patients (76.6%) had complete records available for analysis (records were considered complete if they contained a clear initiating steroid dose and documented inflammatory markers); 83 patients in this group (35.6%) achieved the current recommended diagnostic standard, and 13 (5.6%) achieved the current diagnostic standard, with clear documented evidence of a process of exclusion of other causes of symptoms.

These results are summarised in Table 3.

**Table 3:** Ongoing management of patients with PMR in primary care

**Category**	***n*(%)**
Referred to secondary care	135 (44.4)

**Existing comorbidity**	
Osteoporosis	10 (3.3)
Gastric symptoms	59 (19.4)
Nil	235 (77.3)

**Comorbidity developed**	
Osteoporosis	12 (3.9)
Gastric symptoms	56 (18.4)
Nil	236 (77.6)
Prescribed gastric prophylaxis	85 (28.0)
Prescribed osteoporosis prophylaxis	183 (60.2)

## DISCUSSION

### Summary

While some aspects of the primary care diagnosis and management of PMR, such as initial corticosteroid dose, documentation of typical symptoms, and response to steroids, appear to be in line with national guidance, documentation of other areas of care, for example a thorough process of exclusion of other diagnoses, could be improved. Managing the potential side effects and complications of long-term corticosteroid therapy, specifically gastric symptoms and osteoporosis, does not appear to be routinely considered, especially for gastric prophylaxis, despite the high frequency of complications.^17^

### Strengths and limitations

The major limitation of the present study surrounds its retrospective-records-based nature. It is recognised that every aspect of a consultation cannot be documented in the limited consultation time available, although relevant information may well have been exchanged. This limitation is further compounded by the free-text character limit of 255, as key information may well have been documented but was not available from these databases. Nevertheless, as complete prescription data were available for these patients, it is reasonable to assume that complications requiring a change in treatment will have been included in the study results. It is also recognised that there is the potential that errors in extraction and data re-checking could have occurred, as only a single researcher was used. Additionally, this study was conducted in north Staffordshire, an area that has high levels of deprivation,^22^ which, coupled with local medical guidance, policies, and culture could impact on how generalisable the study findings are.

This study represents one of the largest investigations of PMR in the community, and as such is an important addition to the literature. While there is evidence that GP diagnosis of PMR is not always accurate,^10^ discordance even among experts still exists as to the defining characteristics of the disorder.^13^ Patients included in this study had mean ESR recordings in the range expected for PMR, with age and sex demographics that were classic for the condition. This gives some confidence in the accuracy of the primary care diagnosis. Furthermore, GPs coding these patients thought that they clinically had PMR; therefore management should be in line with accepted best practice for the time, irrespective of the actual diagnosis. Diagnostic accuracy is clearly essential, as misdiagnosis could result in prolonged inappropriate treatment with corticosteroids or a missed opportunity for early treatment of, for example, malignancy. Current diagnostic pathways advise a low threshold for early specialist referral for diagnostic confirmation in patients with normal or very high inflammatory markers, atypical symptoms, poor response to corticosteroids, or prominent systemic features.^15^ Diagnostic accuracy therefore represents a clear area where further primary care-based research is required.

The databases in this study have been validated,^20^,^21^ and the clinicians participating receive regular training and feedback on their clinical coding, providing further confidence in the data.

### Comparison with existing literature

One previously published retrospective primary care-based cohort study included patients consulting between 1994 and 2003.^8^ The present study confirms the findings of this earlier study that around 10% of patients with a diagnosis of PMR have a normal ESR. The rates of referral to secondary care are higher in the present study, which may reflect the characteristics of participating practices or errors in coding referrals in the electronic databases.

### Implications for research and practice

PMR is a disorder that is commonly managed exclusively in general practice, and so future research in PMR needs to include patients recruited from primary care. By 2030, the Department for Work and Pensions estimates that people aged >50 years will comprise almost one-third of the workforce and almost one-half of the adult population,^23^ and so the number of patients with PMR encountered in primary care is set to rise. Accurate diagnosis and evidence-based management is essential to avoid preventable serious side effects and complications of long-term corticosteroid therapy. A heightened awareness of managing potential comorbidity and access to best practice guidelines is essential to optimise care and minimise complications among patients with PMR being managed in the community.
